# High temporal resolution delayed analysis of clinical microdialysate streams†
†Electronic supplementary information (ESI) available. See DOI: 10.1039/c7an01209h


**DOI:** 10.1039/c7an01209h

**Published:** 2018-01-16

**Authors:** S. A. N. Gowers, K. Hamaoui, P. Cunnea, S. Anastasova, V. F. Curto, P. Vadgama, G.-Z. Yang, V. Papalois, E. M. Drakakis, C. Fotopoulou, S. G. Weber, M. G. Boutelle

**Affiliations:** a Department of Bioengineering , Imperial College , London , SW7 2AZ , UK . Email: m.boutelle@imperial.ac.uk; b Department of Surgery & Cancer , Imperial College , London , SW7 2AZ , UK; c Ovarian Cancer Action Research Centre , Department of Surgery & Cancer , Imperial College , London , W12 0NN , UK; d The Hamlyn Centre , Imperial College , London , SW7 2AZ , UK; e School of Engineering and Materials Science , Queen Mary , University of London , Mile End Road , London , E1 4NS , UK; f Department of Chemistry , University of Pittsburgh , PA 15260 , USA

## Abstract

This paper presents the use of tubing to store clinical microdialysis samples for delayed analysis with high temporal resolution, offering an alternative to traditional discrete offline microdialysis sampling. Samples stored in this way were found to be stable for up to 72 days at –80 °C. Examples of how this methodology can be applied to glucose and lactate measurement in a wide range of *in vivo* monitoring experiments are presented. This paper presents a general model, which allows for an informed choice of tubing parameters for a given storage time and flow rate avoiding high back pressure, which would otherwise cause the microdialysis probe to leak, while maximising temporal resolution.

## Abstract 2

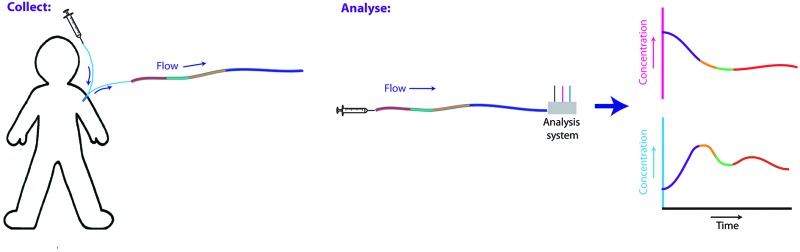
This paper presents the use of tubing to store clinical microdialysis samples for delayed analysis with high temporal resolution, offering an alternative to traditional discrete offline microdialysis sampling. A model allowing optimal results is described.

## Introduction

Microdialysis is an established clinical tissue sampling technique to collect local biomarkers in which a small (500–600 μm diameter) sterile probe is placed in the tissue to be studied. Low flow rates (0.1–2.0 μl min^–1^) through the probe set up a concentration gradient across a semi-permeable membrane, allowing exchange of molecules between the probe and the tissue extracellular fluid. This creates a sterile dialysate stream that can be analysed outside of the tissue. Traditionally this dialysate stream is collected in microvials, typically every 30 min^–1^ hr, for offline analysis. However, online microdialysis has become an important *in vivo* monitoring tool when coupled to high-resolution analytical techniques.^1^–^9^ Continuous analysis with biosensors or rapid online sampling (typically 1 sample per min or faster) has been applied to a wide range of applications as it allows detection of dynamic changes in metabolites^6^–^11^ and neurotransmitters,^12^,^13^ which can provide important real-time information about ischaemia and tissue health. If online analysis is not possible, discrete manual sampling in microvials results in much poorer temporal resolution, which may fail to resolve important clinical events seen with online analysis. For example, in monitoring spreading depolarisations in traumatic brain injury patients metabolic events take place on a 5-minute timescale.^6^ In monitoring free flap surgery metabolic changes following arterial and venous anastomosis, reestablishing blood flow, occur on similar timescale.^7^

Online microdialysis may not be possible for a number of reasons: (a) technical issues occurring with the analysis system. If this occurs during important clinical monitoring experiments, which are rare and therefore of high value, the ability to combine the data with other complete online data sets is lost. (b) Online sampling may need to be carried out in a separate location to the analytical measurement, examples from our work include in an abattoir and in a primary human tissue laboratory. (c) During proof-of-concept experiments in the early stages of a new application, the analysis system may not be optimised for real-time measurement, for example if the analysis time exceeds the time to collect the required sample volume. In this case an online collection system that retains temporal resolution would be highly beneficial.

The concept of storing samples in lengths of fine-bore tubing was first proposed by Linder *et al.* who described the fabrication of ‘cartridges’ of flexible poly(ethylene) tubing containing a sequence of plugs of fluid, which they used to store and deliver reagents.^14^ The authors used air spacers to separate the plugs of reagents and to prevent mixing; they showed that the reagent plugs were unaffected by physical movement. To improve the temporal resolution of offline microdialysis, Wang *et al.* used a similar method to store dialysate samples in segmented flow in fine-bore tubing.^15^ The authors showed that samples collected in this way were stable in the freezer for up to 8 days. However, this was not applied to continuous dialysate streams where mixing across the sample could present more of a challenge. Segmented flow gives improved temporal resolution compared with continuous flow as Taylor dispersion is minimised once segmentation has occurred, however, it is much more complex to achieve and is therefore not always practical, particularly in a clinical setting.

In this paper we use tubing to store dialysate from *in vivo* and *ex vivo* applications for delayed analysis, allowing subsequent high temporal resolution analysis when it would not otherwise have been possible. We consider sample stability and exemplify the use of this methodology in a number of individual online clinical microdialysis experiments spanning a variety of situations from our own work, in which events occur over a range of timescales, to determine the robustness and suitability of the methodology. Finally, we present a model that allows, for future measurements, the optimum choice of tubing size given a required collection time, flow rate and maximum back pressure.

## Materials and methods

### Materials and reagents

Glucose oxidase (GOx) from *Aspergillus niger*, lactate oxidase (LOx) from *Aerococcus viridians* and horseradish peroxidase were purchased from Sekisui Diagnostics. All other reagents were obtained from Sigma-Aldrich. Portex fine-bore polyethylene tubing was purchased from Smiths Medical, UK. Fluorinated ethylene propylene (FEP) tubing was purchased from Microbiotech, UK.

### Storage and delayed analysis of online dialysate samples

Portex tubing (0.4 mm inner diameter, 0.8 mm outer diameter) was used for storing all clinical dialysate samples. At a flow rate of 2 μl min^–1^, 1 m of this tubing stores just over 1 hour of dialysate. During an online experiment the outlet of the microdialysis probe was connected to a length of coiled storage tubing, which had been primed with T1 solution (2.3 mM calcium chloride, 147 mM sodium chloride, 4 mM potassium chloride), using a tubing adapter (Microbiotech AB). The lengths of storage tubing were labelled to record the direction of flow. Once filled, each end of the storage tube was melted with a heated wax pen and the ends were compressed to seal the tubing, taking care to keep the ends level at all times to avoid losing the sample. To analyse the sample the stored dialysate was pumped through the analysis system, effectively flowing the liquid stream as if in real time.

To test the long-term storage of such samples, a microfluidic platform consisting of LabSmith programmable pumps and valves was used to inject a controlled volume of glucose/lactate standards into the tubing, creating identical sample tubes. The system automatically directed glucose/lactate standard into a 1 m storage tube at 8 μl min^–1^ in steps of 0.5 mM, 2 mM and finally 0.5 mM for 5 min each. The storage tubes were sealed and stored in the freezer at –80 °C for 72 days before being analysed using the rapid sampling microdialysis system.

### Discrete and continuous analysis systems

#### Rapid sampling microdialysis analysis system (rsMD)

The rsMD analysis system used here is an example of a relatively high-resolution discrete analysis system, comparable to flow injection, liquid chromatography-mass spectrometry or capillary electrophoresis analysis systems.^3^ In this system a 100–200 nl sample is automatically injected through an enzyme reactor to a downstream electrode, giving a measurement for glucose or lactate every 30 s. The rsMD system has been described previously^7^,^10^ and full details are given in the ESI.†

#### Microfluidic biosensor analysis system

The microfluidic biosensing system used here is an example of a continuous analysis system, such as can apply to optical and electrochemical detection.^16^ This system has been described previously^6^,^9^ and full details are given in the ESI.† Briefly, glucose and lactate biosensors fabricated using combined needle electrodes^17^ and functionalised in three layers^18^,^19^ were positioned in a poly(dimethylsiloxane) (PDMS) microfluidic chip.^20^

### Validation against online method

An experiment was carried out to compare results obtained using the online collection and delayed analysis method with those recorded online. Two microdialysis probes (Microbiotech, 4 mm polyethersulphone membrane length, 0.6 mm outer diameter, 6 kDa cut-off, perfused at 1 μl min^–1^) were positioned close together in the medullary region of a porcine kidney, which had been retrieved from an abattoir and brought immediately back to the lab, as described elsewhere.^10^ The dialysate of one probe was connected to the rsMD system *via* 20 cm of FEP and analysed for levels of lactate in real time. The dialysate from the second probe was collected into a 6 m length of storage tubing (Portex, 0.4 mm inner diameter) and was pumped through the rsMD system at the end of the online experiment.

### *In vivo* and *ex vivo* experiments

Data is presented here from three separate clinical collaborations. Use of patient kidney and cancer tissue samples was approved by the Imperial College Healthcare NHS Trust Tissue Bank. For monitoring of cycling volunteers, all procedures were approved by the local ethics committee (NRES 10/H0808/124, protocol CRO1608), informed consent was obtained and probes were inserted by a qualified clinician. All procedures were carried out in accordance with the Declaration of Helsinki. In all cases the microdialysis probe was perfused with T1 physiological solution. For the cycling experiment sterile solution was purchased from MDialysis AB. All dialysate was stored in Portex tubing, 0.4 mm inner diameter (Smiths Medical, UK).

#### Transplant organs monitoring

For both porcine and discarded human kidneys, a MAB11.35.4 (Microbiotech, 4 mm polyethersulphone membrane length, 0.6 mm outer diameter, 6 kDa cut-off) was used, perfused at 2 μl min^–1^. For the porcine kidney *ex vivo* monitoring experiment, the probe was inserted after 45 min warm ischaemia and remained in place while the kidney was brought to the lab at Chelsea and Westminster Hospital on ice. A 5 m length of storage tubing was used to collect the dialysate and was analysed at a later time following storage at –80 °C. The sample was analysed using the rsMD system. For the discarded human kidney *ex vivo* monitoring experiment, the organ was subjected to 20 min warm ischaemia, approximately 16.5 hours of storage on ice and 5 hours of perfusion with a cold preservation solution, after which it was perfused with oxygenated warm reperfusion solution (Krebs-Henseleit buffer). During the reperfusion phase the dialysate from a microdialysis probe positioned in the cortex was analysed in real time for levels of glucose and lactate using the rsMD analysis system. The dialysate from a second probe placed in the medulla was collected into a length of storage tubing (8 m) and was analysed at 2 μl min^–1^ for levels of glucose ad lactate using the rsMD system at a later time following storage at –80 °C.

#### Cyclist monitoring

To measure subcutaneous levels of glucose and lactate in cycling athletes, a sterile CMA 63 (MDialysis AB, Sweden, 10 mm polyarylethersulphone membrane, 0.6 mm outer diameter, 20 kDa cut-off) was inserted subcutaneously into the lower back as described elsewhere.^9^ The probe was perfused at 1 μl min^–1^ and the dialysate was collected into a 0.36 m length of storage tubing. Following collection, the sample tubes were sealed and stored in the freezer. The samples were run at a later time and analysed for levels of glucose and lactate using the rsMD system.

#### Metabolite measurement in ovarian cancer samples

A CMA 12 microdialysis probe (Linton Instruments, 4 mm polyarylethersulphone membrane, 0.5 mm outer diameter, 20 kDa cut-off) was chosen for its rigidity. It was inserted into a piece of cancerous omentum tissue, which had been surgically removed from a patient undergoing primary cytoreductive surgery for advanced ovarian cancer. The probe was perfused at 1 μl min^–1^ and the dialysate was collected into a 0.75 m length of storage tubing in a sterile tissue culture hood at Hammersmith Hospital campus. The sample tubes were sealed and brought back to the lab at the South Kensington campus where they were frozen until analysis for levels of glucose and lactate using the microfluidic biosensor system.

## Results and discussion

### Storage conditions

Dialysate samples stored in tubing were stable at room temperature for up to 24 hours. After this time, they were stored in the freezer to prevent possible degradation of the sample. To test that temporal resolution was retained and that there was minimal sample degradation using this storage method, one-metre lengths of storage tubing were filled with glucose/lactate standards in 3 sections of 0.5, 2 and 0.5 mM using the LabSmith microfluidic system described above.

**Fig. 1:**
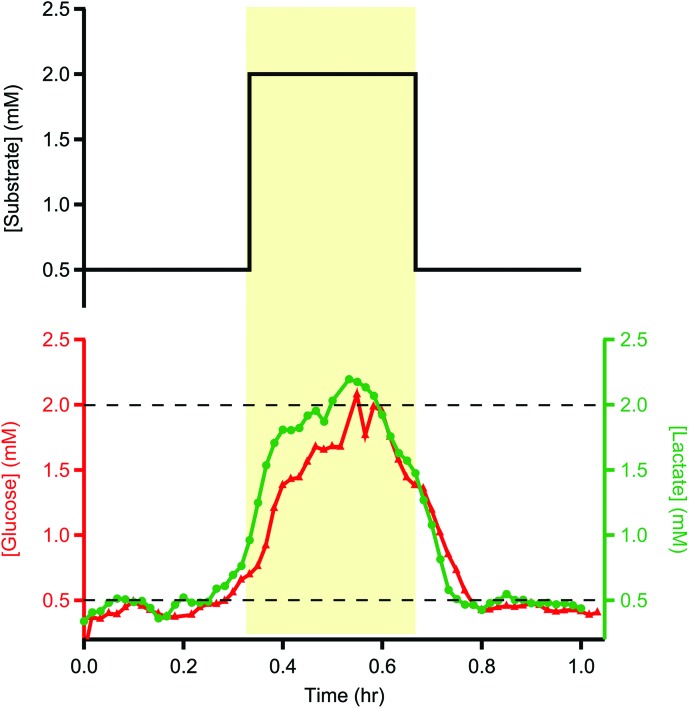
Glucose (red triangles) and lactate (green circles) levels measured in storage tubes filled with 20 min pulses in concentration of 0.5, 2 and 0.5 mM. Storage tubing was kept in the freezer for 72 days at –80 °C. The samples were analysed at 2 μl min^–1^ using the rsMD system, giving a reading every minute for each metabolite. The dotted lines indicate the concentrations used to fill the tubes. The top trace indicates the input concentrations.

The sample tubes were stored in the freezer at –80 °C for 72 days before being analysed for glucose and lactate using the rsMD system described above. Fig. 1 shows example data for a sample tube analysed for glucose and lactate.

Degradation of the sample seems to be minimal. Some temporal spreading of concentration changes occurred, as expected due to analyte dispersion during flow into and out of the tube. Despite this, the 20 min pulse in concentration can still be resolved both in terms of timing and magnitude, demonstrating that long-term storage of these samples is possible. The exemplar traces shown here are representative of other traces recorded in the same way.

### Validation against online measurement

**Fig. 2:**
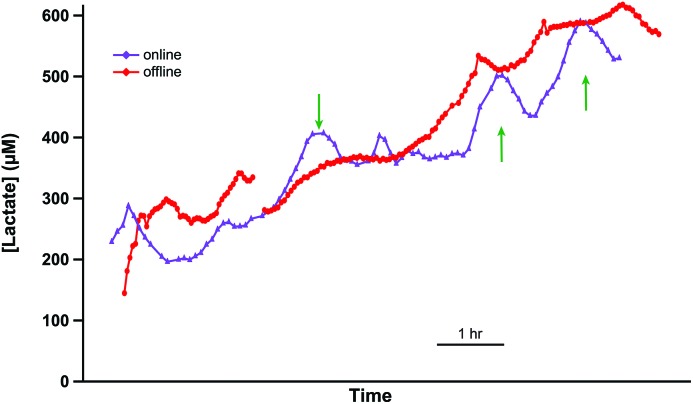
Validation of collection in storage tubing for delayed analysis. The purple (triangles) trace shows the dialysate lactate concentration analysed online using rsMD. The red (circles) trace shows the lactate level in the dialysate from a second microdialysis probe, which was collected in storage tubing and analysed offline at a later time using rsMD. Results have been time-aligned. Green arrows indicate points at which the offline data has been attenuated due to dispersion.

In order to validate this methodology *in vivo*, it was important to compare levels detected by collecting and storing dialysate in tubing with those detected online. To achieve this, lactate levels were measured in the medulla of a cold-perfused porcine kidney *ex vivo* both online and using the online collection and delayed analysis method. Two microdialysis probes were inserted close together in the same region of the kidney and were perfused at 1 μl min^–1^. One dialysate stream was analysed online in real time using the rsMD analysis system. This is shown by the purple trace in Fig. 2. Dialysate from the second probe was collected into a length of storage tubing (6 m). This stored dialysate was then also analysed using the rsMD analysis system, shown in Fig. 2 red trace.

The two traces show very similar overall trends. There is some evidence of analyte dispersion attenuating the offline data (red trace) response to faster changes, examples indicated by green arrows. However, the mean difference between the levels measured at each time point using the two methods was 25 μM, relatively small compared to the magnitude of the levels detected. This demonstrates that tubing storage is a viable alternative to traditional discrete sample collecting, for a given temporal resolution, when online analysis is not possible.

### High temporal resolution analysis

For a given injection-based online analysis system the temporal resolution is limited either by the time for sample analysis or by the time taken to accumulate a sufficient sample volume in the sample loop to exceed the mass sensitivity limit (determined by product of sample volume and analyte concentration).^3^ The temporal resolution is maximal when these are *equal*. This could be achieved in an online system by optimising the sample loop volume, hence varying the fill time. However, in practice, particularly with low-volume internal loops typically favoured by online analysis systems, this is not possible. Storing the liquid stream in tubing provides a useful alternative strategy.

An advantage of having separate collection and analysis flow rates is that the microdialysis flow rate and analysis flow rate can be optimised independently (explored further in ESI, Fig. S1†). For example, in ischaemic tissue lactate levels can be very high and beyond the linear range of the assay. By using a high microdialysis flow rate we can reduce the microdialysis probe efficiency, hence lowering the dialysate lactate concentration to within the assay working range. Subsequently, the collected sample can be analysed with a sufficiently sensitive online instrument at a low flow rate for high time resolution.

The converse is also true where tissue levels are low. We can slow the collection flow rate to increase the probe recovery efficiency from the tissue. Analysis then takes place at a faster flow rate so that the loop fill time is not too long. Ultimately, both approaches are limited by the mass sensitivity of the assay. An alternative approach, only possible with storage tubing, is to use a computer-controlled syringe pump to fill the loop at a high flow rate then pause while analysis takes place. This minimises waste of precious samples.

### Examples of use in various *in vivo* and *ex vivo* applications

**Fig. 3:**
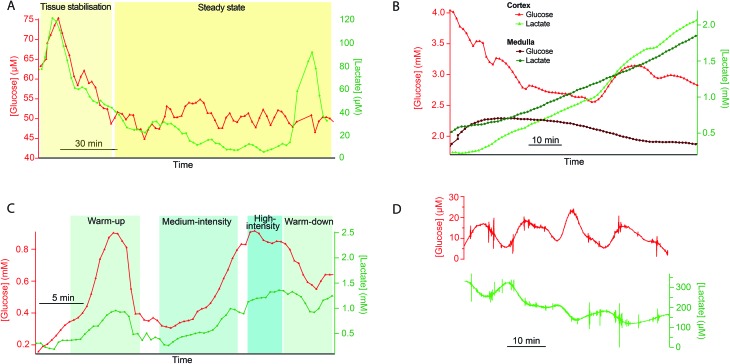
Examples of tissue measurements made using online collection into storage tubing followed by delayed analysis. A. Glucose (red, triangles) and lactate (green, circles) levels measured in dialysate collected from a porcine kidney soon after retrieval (warm ischaemia time 45 min) into a length of storage tubing (5 m) as it travelled from the abattoir to the lab. The microdialysis probe was perfused at 2 μl min^–1^. Dialysate was analysed at 4 μl min^–1^ using rsMD at a later time as the analysis system was not portable. This gave a point every minute for each metabolite, corresponding to a point every 30 s in real time. B. Dialysate glucose (red) and lactate (green) levels measured in two regions of a discarded human kidney during warm reperfusion. The microdialysis probe in the cortex (triangles) was perfused at 2 μl min^–1^ and analysed in real time using rsMD. The second microdialysis probe in the medulla (circles) was perfused at 2 μl min^–1^, collected into a length of storage tubing (8 m) and run through the rsMD system at 2 μl min^–1^ at a later time (with a point every minute for each metabolite) as there were not enough analysers to measure glucose and lactate in both dialysate streams in real time. C. Glucose (red, triangles) and lactate (green, circles) levels measured in dialysate collected subcutaneously from a cyclist during varying levels of exercise intensity. The microdialysis probe was perfused at 1 μl min^–1^ and collected into storage tubing (0.36 m) and analysed using the rsMD system at 0.5 μl min^–1^, with a point every minute for each metabolite, corresponding to a point every 30 s in real time. D. Glucose (red, top) and lactate (green, bottom) levels measured in dialysate collected from a sample of cancerous omentum tissue removed from a patient with advanced ovarian cancer. The microdialysis probe was perfused at 1 μl min^–1^, collected into a length of storage tubing (0.75 m) and run through a microfluidic chip housing glucose and lactate needle biosensors at 1 μl min^–1^.

This method of online collection into lengths of storage tubing followed by delayed analysis has proven useful in many different microdialysis monitoring applications within our group. In particular it has proven crucial during high-value experiments when technical issues occur with the analysis system so that important data from one-of-a-kind monitoring experiments can still be collected.^7^ Further examples to illustrate the wide range of uses to which this methodology can be applied are shown in Fig. 3.

#### Monitoring transplant kidneys

Our aim was to monitor metabolite levels in transplant kidneys after retrieval in order to assess their viability for transplantation. As the rsMD analysis system is large and not portable it was only possible to monitor the organs once they were brought to the lab. In a proof-of-concept experiment, using pig kidneys, to collect data from the crucial time period immediately after the kidney had been retrieved, a microdialysis probe was inserted into the kidney at the abattoir and a length of storage tubing was attached to the probe outlet. Once back at the lab, the probe was connected to the online analyser to provide real-time readings and the sample tubes were sealed and stored in the freezer for delayed analysis. Example data of dialysate glucose and lactate levels for a porcine kidney during this initial period after organ retrieval are shown in Fig. 3A. The initial peak seen in both glucose and lactate levels is likely due to tissue stabilisation following probe insertion, which typically occurs after 10–15 minutes.^8^ Levels of both glucose and lactate were found to be low compared to levels measured previously at a later point after organ retrieval.^10^ Metabolite levels remained low and generally stable after the initial stabilisation period, with the exception of a peak in lactate concentration after 2.5 hours of monitoring.

As part of this project, working this time with discarded human kidneys, we were interested in measuring levels in both the cortex and the medulla regions of the kidney in order to obtain a more complete picture of the health of the organ. However, it was only possible to measure levels of glucose and lactate in one dialysate stream with the rsMD analysis system. Therefore, dialysate from a second probe, placed in the medulla was collected into a length of storage tubing for delayed analysis. Dialysate glucose and lactate levels in both the cortex (online measurement) and medulla (delayed analysis) are shown in Fig. 3B for a discarded human kidney.

#### Monitoring exercising cyclists

This study aimed to develop a wearable biosensing system to monitor subcutaneous energy metabolite levels in cycling athletes.^9^ As a first step towards this goal we used delayed analysis of samples collected online in storage tubing to define the scope of the analytical problem. This approach allowed cycling trials to be carried out without the complication of connecting the probe to a large clinical trolley during exercise, enabling us to simply ascertain the feasibility of monitoring tissue metabolite levels during cycling as well as the typical magnitude and timescale of any changes for design of the new analysis system. It also allowed us to optimise the experimental protocol for future experiments independently of the analysis system. Fig. 3C shows subcutaneous dialysate glucose and lactate levels in a cyclist undergoing varying levels of cycling intensity measured during online collection and delayed analysis. Dynamic changes in the metabolite levels during varying cycling intensity can clearly be detected using this methodology.

#### Measuring metabolite levels in ovarian cancer samples

In this study we were interested in establishing for the first time metabolite levels in samples of omentum tumour tissue surgically removed from patients with advanced ovarian cancer. As this was a new application, collecting dialysate samples online into sample tubes and storing them for delayed analysis in the lab allowed us to collect data immediately while the protocol was still being optimised and without needing to set up the online system in a biological safety cabinet where space is limited and sterility is crucial. The tubing allowed the low sample volumes (30 μl for entire analysis time) to be handled easily and with less risk of losing irreplaceable data. Fig. 3D shows dialysate glucose and lactate levels measured in a sample of cancerous omentum tissue. It is possible to resolve small fluctuations in concentration in both metabolites using this method.

### A general model for tubing storage of dialysate

**Fig. 4:**
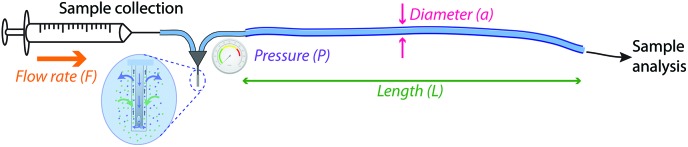
Schematic representing the factors that can influence temporal resolution and pressure (*P*): inner diameter (*a*), volumetric flow rate (*F*) and tubing length (*L*). Inset: Schematic of diffusion across microdialysis membrane.

The experiments presented were carried out using readily available tubing with a very cautious perspective on creating back pressure. However, after the fact, we were curious as to what the actual best experimental conditions were for these situations. In order to optimise the temporal resolution of this method for different clinical monitoring situations we have devised a model that describes how to choose the tubing parameters, diameter and length, to achieve a particular objective, assuming that the tubing is first primed with solution. Fig. 4 illustrates the factors that can affect pressure (*P*) and temporal resolution when collecting microdialysate, (i) tubing inner diameter (*a*) (ii) volumetric flow rate (*F*), and (iii) tubing length (*L*). These calculations assume that the flow rate for collection and analysis is the same and that there is no solute diffusion during static storage at –80 °C (justified in ESI†). The basic idea is that there are seven inter-related parameters. These are tabulated below (Table 1).

**Table 1:** Definitions of variables and parameters

Parameter	Symbol	Units
Tube length	*L*	m
Tube radius	*a*	m
Collection time	*t*_s_	s
Fluid velocity	*V*	m s^–1^
Fluid flow rate	*F*	m^3^ s^–1^
Pressure	*P*	Pa
Variance	*σ*_*t*_^2^	s^2^

One is permitted to choose no more than three of these parameters freely. The others are functions of the chosen three. There are several different scenarios justifying the chosen three parameters. Determining the remaining four parameters is a matter of algebra. One example follows, and then the results, but not the derivation, are shown for several other scenarios.

The researcher may be required to use a fixed *flow rate* (*F*) and may have a particular piece of tubing with a certain *length* (*L*) and *radius* (*a*) in hand. What is the total collection time, the variance, and the pressure developed? (It is most convenient to use flow rate to describe the rate of fluid flow because this is a common microdialysis parameter. We will not use fluid velocity to describe the rate of fluid flow except transiently where it is convenient for derivations. Thus, we focus on six of the seven parameters.)

The Poiseuille equation describes the pressure:1
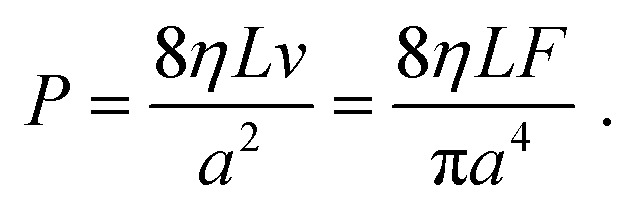



It turns out to be convenient to define a parameter, *τ*, which arises in derived quantities. We assume a constant viscosity here, so *τ* is determined by the pressure and has units of time (s).2
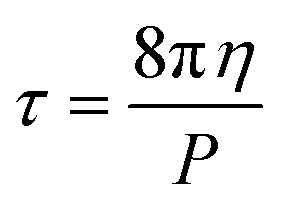



The term *τ* will often appear in equations below rather than pressure explicitly being shown.

The collection time is the ratio of tube volume to flow rate shown in eqn (3).3
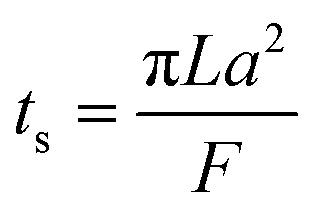



A hypothetical “spike” in concentration entering the tube will exit the tube as a Gaussian peak with a variance, eqn (4). The square root of this is the standard deviation, which ultimately defines the time resolution of the measurements carried out on the contents of the tube. The first term inside the brackets of eqn (4) is due to axial convective dispersion relaxed by radial diffusion first elucidated by Taylor.^21^ The second term is due to axial diffusion as elucidated first by Aris.^22^ The second term only comes into play for long times/low flow rates.4
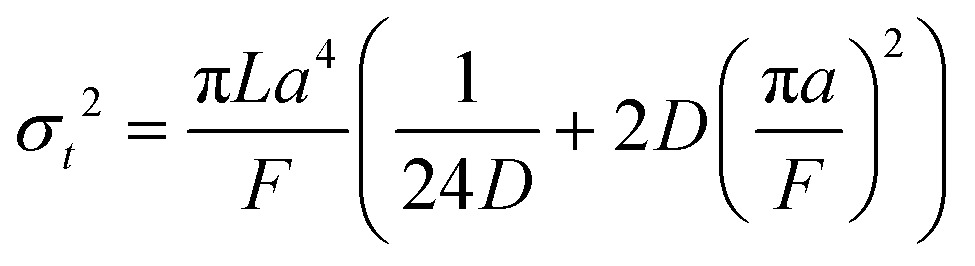



Note that choosing the parameters *L*, *a*, and *F* completely determines the parameters of the collection: *P*, *t*_s_, and *σ*_*t*_^2^. In this hypothetical spike, the rise time (10–90%) exiting the tubing is equal to 2.56*σ*. This and other scenarios are shown in Table 2 below. This table is not exhaustive – there are many more combinations of three parameters chosen by the user that define the remaining three (twenty in total). But the sampling below is instructive. Row 1 illustrates the case just derived above. Here, the scenario is dictated by the tubing itself (*L*, *a*) plus a flow rate, which has been established for the microdialysis portion of the experiment. In rows 2 and 3, the flow rate is again fixed as it may be in many labs with experience in microdialysis. The tubing radius is also fixed and one other operational parameter is fixed: *P* or *t*_s_. In rows 4–6, on the other hand, neither the tubing length nor the radius is specified. These rows focus more on the *figures of merit* of the sample collection, namely *t*_s_ and *σ*_*t*_^2^, plus parameters of the microdialysis, *F* and *P*. The pressure maximum is an important criterion because the microdialysis membrane itself will pass fluid under pressure. Pressure inside the probe is developed by fluid flow downstream of the probe passing through the collection tubing. As a result of this pressure two things occur: the probe volume expands and the rate of fluid flow through the membrane into the tissue is increased.^23^–^25^ This problem has been addressed by Kiritsis.^26^ He determined quantitatively the effect of pressure on the microdialysis probe itself. The data obtained by Kiritsis were for a 13 kDa regenerated cellulose-based probe from Spectrum, Inc., Laguna Hills, CA, USA. This molecular weight cut-off is commonly used in microdialysis measurements of low molecular weight solutes such as metabolites, ions, and many neurotransmitters.

**Table 2:** Equations to derive three of six experimental parameters from the three chosen by the experimenter. The quantities with an asterisk are asserted by the experimenter. The remaining three quantities arise from those as shown

	*F*	*P*	*a*	*L*	*t*_s_	*σ*_*t*_^2^
1	*	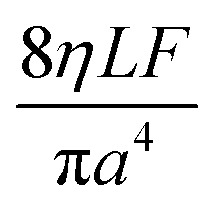	*	*	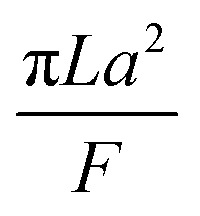	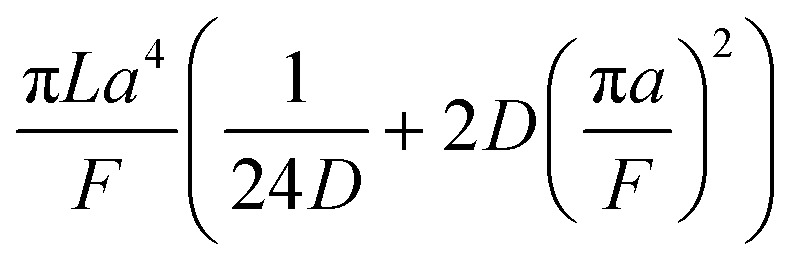
2	*	*	*	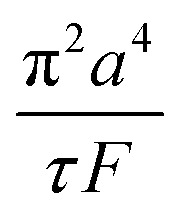	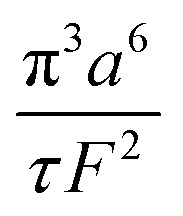	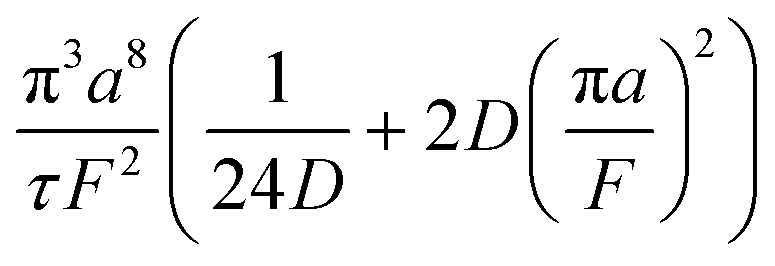
3	*	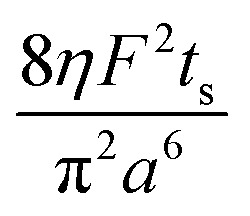	*	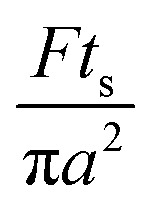	*	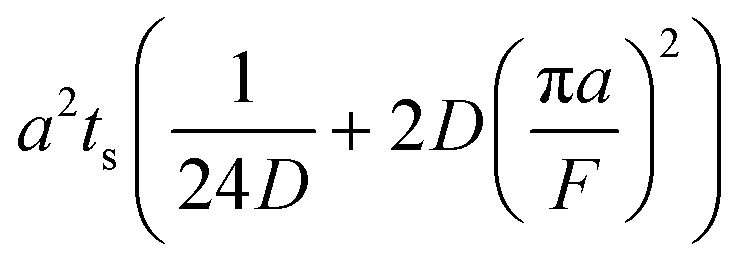
4	*	*	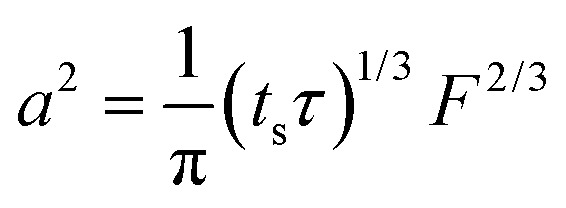	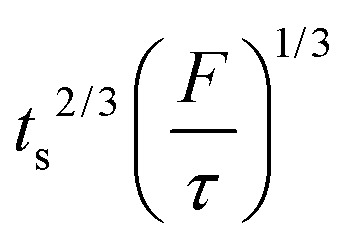	*	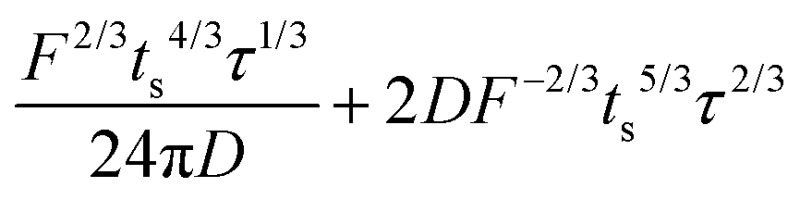
5^*a*^	*	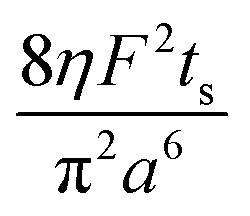	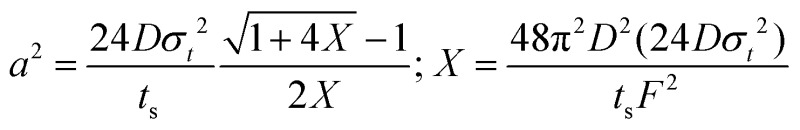	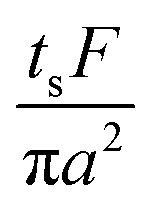	*	*
6^*a*^	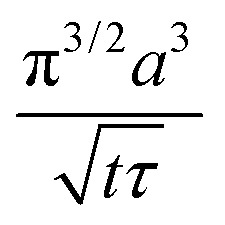	*	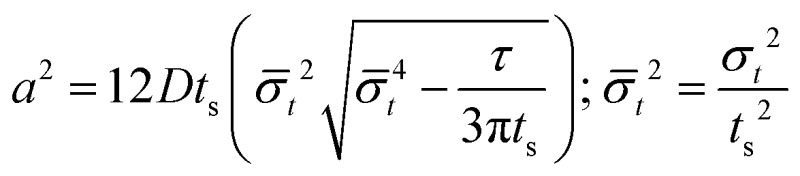	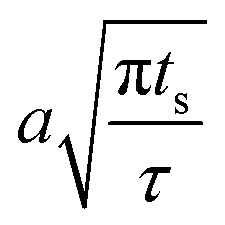	*	*

^*a*^Note that in the last two rows, explicitly showing calculated quantities as a function of the three chosen parameters becomes cumbersome. Thus, when two of the three provided parameters are *σ*_*t*_ and *t*_s_ it is most efficient to calculate the radius, *a*, and then calculate the other parameters as a function of *a*.

We can use Kiritsis’ data to determine a suggested pressure maximum for a microdialysis probe. This pressure maximum will limit the combination of collection tubing length, radius, and the flow rate. Somewhat arbitrarily, we have chosen here a maximum pressure of 50 kPa, or about 0.5 atm. At this pressure, the probe material expands by a mere 4% and the fluid flow through the membrane into the tissue is but 3 nl min^–1^ (see ESI† for the latter calculation). A simple calculation shows that the velocity of the advancing fluid “front” resulting from this leakage has a minimal impact on collection efficiency because solute transport into the probe by diffusion through the leaking fluid is at a much higher velocity than the front's opposing flow.

Now, let us look at some actual scenarios based on specifying certain quantities to set up an experiment in microdialysis collection (Table 2). The first scenario is based principally on what the laboratory capabilities and practices are. This is exemplified by rows 1 and 2 in the table.

The microdialysis flow rate is fixed (1.0 μl min^–1^), you have tubing of a particular radius (100 μm), and you want to be conservative regarding the pressure to which the probe is exposed (no more than 1 kPa). What can be accomplished (this corresponds to row 2)? According to the calculations, using the maximum allowable pressure would permit a collection time of 83 minutes (1.38 hours) with a 2.65 m long tube resulting in a *σ*_*t*_ of 59 s. Using only half the allowable pressure would decrease length and time by a factor of 2 and the value of *σ*_*t*_ to 42 s, a decrease of 
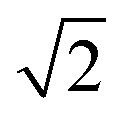
. Decreasing the flow rate to 0.3 μl min^–1^ would permit collection for 15.4 hours in a tube 8.82 m long with the same radius. The resulting *σ*_*t*_ of 203 s is long, but a small sacrifice to make for the long collection time.

As in the case of chromatography, performance increases with increased pressure. The meaning of “performance” in the current context could be the time resolution, the collection time or some combination of these. Thus it is useful to explore what could be accomplished by working at the pressure limit. What is the outlook for a better collection time with *σ*_*t*_ = 59 s and *F* = 1.0 μl min^–1^ as above when working at 50 kPa? One could achieve 3.7 h of collection with a *σ*_*t*_ of 59 s, considerably better than the scenario above (length = 18.8 m; tubing radius = 61 μm). A 15.4 hours collection with a flow rate of 0.3 μl min^–1^ can be accomplished at *P* = 50 kPa with *σ*_*t*_ of 103 s (length = 32.5 m, radius = 52 μm).

The calculations based on the equations in Table 2 are straightforward using a spreadsheet (; http://stephen-weber-chemistry.squarespace.com/useful-links/). However, it is difficult to get the larger picture of the behaviour of this sample collection approach this way. Earlier, based on the work of Carr *et al.*,^27^ we developed a type of plot to optimise chromatographic conditions.^28^ Here, we have developed analogous plots that incorporate all six parameters for the two types of scenarios: in one the axes of the plots are laboratory parameters, namely tubing *length* and *radius*. In the other, the axes are figures of merit, *time resolution* and *collection time*.

**Fig. 5:**
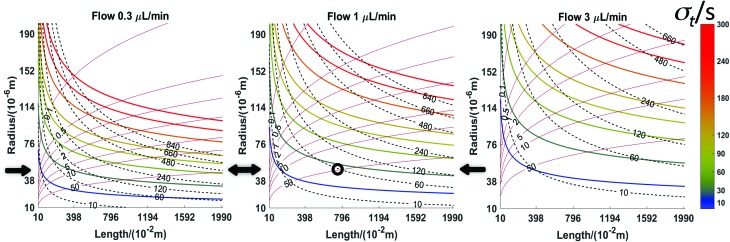
Three laboratory parameters are chosen: tubing length and radius plus flow rate. The former two are the axes and the flow rates of each plot are indicated. Contours of the remaining three parameters comprise each plot. Coloured lines are *σ*_*t*_ (Note colour key at right. Individual contour lines correspond to the numerical values on the colour bar, *e.g.*, the gold line fifth from the bottom is the 120-minute line.), red dashed lines are collection time (minutes) and grey lines are pressures (kPa). Note further the annotations in the left plot. Other parameters: diffusion coefficient of analytes: 6.0 × 10^–10^ m^2^ s^–1^; viscosity: 0.00089 Pa s.

Fig. 5 shows a set of three plots differing by the flow rate. The axes are the tubing radius and length. Contour lines are for three quantities resulting from the choices of flow rate, radius, and length (indicated by text in the left plot): the collection time (dashed red, min), the value of *σ*_*t*_ (coloured lines, s, scale to right), and the pressure (solid black lines, kPa). The plots indicate what the effect of changing a given parameter is. For example, following any individual isobar from left to right shows that longer times result but with a sacrifice in time resolution. Following a particular time line (dashed red) shows that maintaining a constant time for a given flow rate requires length and radius to change. Note that the best time resolution for a given isochrone is to the right, but travelling along an isochrone from the upper left to the lower right is accompanied by an increase in pressure. For any given length and radius, considering all three plots at a single “*x*, *y*” coordinate shows that increasing flow rate decreases sampling time and improves time resolution while increasing pressure. A specific question might be what the effect of flow rate is when using available tubing of a particular radius, *e.g.*, 50 μm (horizontal black arrows in Fig. 5). Moving horizontally along a constant radius line, increasing length, increases collection time, *σ*_*t*_, and pressure. With a microdialysis flow rate of 1 μl min^–1^, this trajectory crosses the 50 kPa contour when collection time is about one hour and *σ*_*t*_ is only 25 s. If the microdialysis flow rate is decreased to 0.3 μl min^–1^ the same collection time and *σ*_*t*_ are achieved at a far lower pressure (less than 5 kPa) with a length of 2.25 m. While resolution suffers, a 20 m length offers 8.7 hours of collection (*σ*_*t*_ = 74 s). At 3 μl min^–1^, this radius is not very useful.

**Fig. 6:**
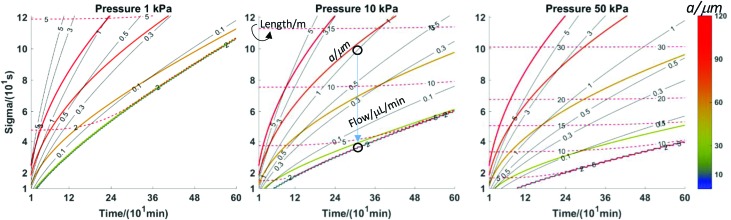
One laboratory parameter, pressure, is chosen. Also chosen are two figures of merit: *σ*_*t*_ and collection time. Contours of the remaining three parameters comprise each plot. Coloured lines are column radius, a (μm). (Note colour key at right. Individual contour lines correspond to the numerical values on the colour bar. The blue line does not appear in this set of plots.) Red dashed lines are length (m) and grey lines are flow rates (μl min^–1^).

Fig. 6 shows another set of plots differing by allowed pressure. On these plots (see annotations on the centre plot), the coloured contours are the tubing radius (μm), the red dashed lines are length (m) and the black lines are the flow rate (μl min^–1^). The axes are the figures of merit, *σ*_*t*_ and collection time. Of course, one would like the former to be low and the latter to be high. This implies that the best results are obtained by conditions at the lower right of the plot. The large, empty space at the lower right corresponds to an inaccessible set of conditions. The limitation is the dispersion of the analytes during flow. Taylor–Aris dispersion cannot be avoided in the apparatus described here. The inaccessible zone corresponds to analyte dispersion being *less* than that due to axial diffusion alone, which is of course impossible. With respect to the inaccessible zone, note that it is smaller at higher pressure. In other words, higher pressure opens up the possibility to improve the figures of merit. Also, for a given time resolution, the longest collection time possible is at the line separating the inaccessible zone from the accessible zone; the same is true for attaining the best time resolution for a given collection time. Thus, to achieve these conditions, length, flow rate and tubing radius should be chosen to be on this line. As a practical matter, the flow rates required are less than 100 nl min^–1^, which is lower than that in most microdialysis measurements. This has practical disadvantages, such as taking significant time for flow to reach steady state after turning on the pump and the near impossibility of finding a leak in the system based on visually observing the leaked fluid. On the other hand, pumps capable of such low flow rates are readily available and relative recovery will increase. An example at a pressure of 10 kPa will demonstrate the advantages. Consider the middle panel in Fig. 6. The black circle near the top indicates an experiment done at the convenient flow rate of 1 μl min^–1^ with a length (12.9 m) and tubing radius (86 μm) suitable for a 5-hour collection time and 10 kPa of pressure. The value of *σ*_*t*_ is 96 s. Now, if the experiment is done for the same 5 h collection time, but at the optimum conditions (shown by the arrow and lower black circle), the flow rate will be 27 nl min^–1^, length will be 3.9 m, the tubing radius will be 26 μm and the value of *σ*_*t*_ will be a much better 36 s. At this pressure, leakage through the membrane would be equal to or less than 1/5^th^ of the 3 nl min^–1^ value calculated for a 50 kPa pressure: 600 pl min^–1^ or 2.2%.

Consider two examples from Fig. 3A and D. Fig. 3A collection was for a much longer time than Fig. 3D (5.2 h *vs*. 1.6 h for complete filling of the collection tube). The circumstances of Fig. 3A are length *L* = 5 m, flow rate = 2 μl min^–1^, *a* = 200 μm. Calculations show that this would have generated less than 300 Pa at the probe and created a calculated *σ* of almost four minutes (230 s). If we had permitted ourselves to develop a pressure of 50 kPa (1/2 atm), Fig. 6 shows that a flow rate of 0.5 μl min^–1^ would dictate a capillary with *a* = 52 μm and length 18.7 m. The resulting *σ* is 59 s. A lower value, 35 s, is possible (*L* = 10.9 m, *a* = 30 μm, *F* = 0.1 μl min^–1^). The pressure experienced by the probe for the conditions of Fig. 3D is 20 Pa, while the calculated *σ* is 127 s (collection time = 96 min, *L* = 0.75 m, *F* = 1 μl min^–1^, *a* = 200 μm). With a pressure of 50 kPa (1/2 atm), Fig. 6 shows that for a time of 96 minutes with a length of 5 m (red dashed line) the flow rate would be 0.1 μl min^–1^ and the radius is less than 30 μm. The resulting *σ* is well under 20 s. The spreadsheet based on the calculations in Table 2 (link above) clarifies that *a* = 25 μm and *σ* = 16 s. This is a considerable improvement in time resolution.

It is appropriate to consider the effect of a particular value of *σ* on the information content arising from the analysis. Under the conditions existing in a sample collection carried out using an open tube, it is safe to assume that the diffusion coefficient of a solute is independent of the concentration of the solute. In such a system, the convolution theorem applies.^29^ An infinitely sharp spike of the concentration of an analyte at the dialysis probe would enter the sensor as a Gaussian profile with a certain standard deviation as discussed above. The time resolution can be thought of as follows. What would the time separating *two* successive analyte spikes of equal intensity need to be in order to perceive them as two individual events as they exit the collection tube? This is analogous to the resolution problem in chromatography in which resolution is related to the separation in time of the peak maxima and the peak width:5
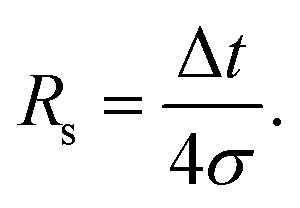



It is plainly evident that a pair of peaks is actually a pair, not a single peak, if *R*_s_ is 0.6 or greater.^30^ Rearranging eqn (5) with *R*_s_ equal to 0.6 leads to a criterion that the time resolution according to the stated criterion is 2.4*σ*.

One also may be interested in a different sort of time resolution: to what degree of accuracy can the time of an event be established? Here, the value of *σ* is much less critical. As the dispersion occurring in the tube is symmetrical (a spike becomes a Gaussian) there is in principle no effect of *σ* on establishing when an event occurred, be it a transient like a spike or peak, or a step up or down. As a practical matter, a large *σ* weakens the signal from the sensor, so establishing the time of an event may be more difficult when *σ* is large because of the erosion of the signal-to-noise ratio.

## Conclusions

We have demonstrated that dialysate can successfully be collected into lengths of storage tubing for delayed analysis while still retaining important temporal resolution. We have also presented a general model that relates key experimental parameters and analytical figures of merit.

Although it is clearly preferable to carry out measurements in real time, this is not always possible and this method offers a promising alternative to traditional offline microdialysis. This methodology provides a means of collecting data with good temporal resolution in experiments with living tissue/human subjects that are carried out away from the lab and allows conditions for collection and analysis to be optimised independently of one another, improving the likelihood of successful data collection. Storage of time-resolved dialysate samples for delayed analysis is particularly useful in the early stages of novel pioneering experiments where it is initially difficult to collect data; in this way the collection protocol can be optimised before online analysis is incorporated. This analysis method could be used in a wide variety of applications, offering an important improvement on traditional offline microdialysis.

Our model clearly suggests that microdialysis users should not be afraid of back pressure, but should quantify pressure limits and then work just below this limit for optimised performance. It is also clear that microdialysis is optimally carried out at low-volume flow rates in probes with small internal dimensions, such as those found in recent micro-fabricated probes.^31^,^32^

## Author contributions

S. G. designed and carried out the experiments, and wrote and revised the manuscript. S. W. devised the model, and wrote and revised the manuscript. M. B. designed and directed the experiments, and wrote and revised the manuscript. K. H. and V. P. designed and contributed to experiments presented in Fig. 2, 3A and B, and reviewed the manuscript. P. C., E. D. and C. F. designed and contributed to experiments presented in Fig. 3D, and reviewed the manuscript. S. A., V. C., P. V. and G.-Z. Y. designed and contributed to experiments presented in Fig. 3C.

## Conflicts of interest

There are no conflicts to declare.

## Supplementary Material

Supplementary information
