# Supplementary material for: High temporal resolution delayed analysis of clinical microdialysate streams
Source: Analyst. 2018 Jan 16;143(3):715–24. doi: 10.1039/c7an01209h (PMC5804479; doi:10.1039/c7an01209h)
Supplement: Supplementary file 1 [file AN-143-C7AN01209H-s001.pdf]

Electronic Supplementary Material (ESI) for Analyst.
This journal is © The Royal Society of Chemistry 2017

Supplementary Information

High temporal resolution postponed analysis of clinical microdialysate streams

S. A. N. Gowers, K. Hamaoui, P. Cunnea, S. Anastasova, V. F. Curto, P. Vadgama, G-Z. Yang, V. Papalois, E. M. Drakakis, C. Fotopoulou, S. G. Weber and M. G. Boutelle

Rapid sampling microdialysis analysis system (rsMD)

The rsMD analysis system can be set up in two configurations, either to analyse two analytes in one dialysate stream or one analyte in two dialysate streams and has been described elsewhere.[1,2] Briefly, in the former case, the dialysate stream is connected to a 100-200 nl sample loop of a custom-made 6-port valve (Valco, Switzerland). A high-performance liquid chromatography (HPLC) pump flows a filtered ferrocene mediator solution into a T-connector at 200 μl/min, splitting the flow evenly between two analysis loops of the valve. Every 30 s a dialysate sample is alternately accelerated into one of the two analysis streams, through either a lactate or a glucose assay, each containing two membranes, the first loaded with substrate oxidase (glucose oxidase for glucose and lactate oxidase for lactate) and the second with horseradish peroxidase (HRP), to a downstream electode, which detects a reduction current. In the configuration in which one analyte is detected in two dialysate streams, each dialysate sample is connected to a separate sample loop of the flow injection valve, either side of the analysis loop. The ferrocene mediator solution is pumped by an HPLC pump into the analysis loop at 100 μl/min. Every 30 s a dialysate sample is injected through the lactate assay to a downstream electrode, alternating between the two dialysate streams. In both configurations, data were collected using a PowerLab data acquisition unit and LabChart software (ADInstruments, Australia) running on a Macbook Pro portable computer (Apple Computers, CA).

Microfluidic biosensor analysis system

The microfluidic biosensing system has been described previously.[3,4] Briefly, it consists of glucose and lactate biosensors housed in a microfluidic chip. Glucose and lactate biosensors were fabricated using combined needle electrodes, made by threading a 50 μm polytetrafluoroethylene (PTFE) insulated platinum/iridium (90%:10%) wire (Advent Research Materials, UK) and a 50 μm polyester insulated silver wire (Goodfellow, UK) through a 27G hypodermic needle.[5] The insulation was removed from the ends of both wires using a flame and the exposed metal was connected to an electrical wire using conductive silver epoxy (RS Components, UK). The needles were filled with epoxy resin (CY1301 and HY1300, Robnor resins) to secure the wires. Once cured, the needles were polished with sandpaper (Buehler, UK) to give a flat surface creating platinum and silver disc electrodes and then sequentially with alumina slurries (1, 0.3 and 0.05 μm). The 50 μm platinum disc formed the working electrode and the silver wire was chloridised to give a 50 μm disc Ag|AgCl reference electrode by dipping the needle tip into potassium dichromate reference solution (BASi, US) for 3s and then into hydrochloric acid (37%) for 20 s to remove the oxide layer from the working and auxiliary electrodes. The stainless steel needle shaft served as the counter electrode. Cyclic voltammetry was used to assess the quality of the working electrode surface. The electrodes were then functionalised in three layers. Initially, the working electrode was coated with poly(m-phenylenediamine) (m-PD) using electropolymerisation to block potential interferences. To do this the needle was placed in a 100 mM solution of m-phenylenediamine in 0.01 M PBS at pH 7.4. The potential was held at 0 V for 20 s, 0.7 V for 20 min to initiate polymerisation and 0 V for 5 min. After electropolymerisation the electrode was rinsed gently with deionised water and cyclic voltammetry was used to verify that the working electrode had been successfully coated. The second step involved dipping the needle tip into a hydrogel layer containing either glucose oxidase or lactate oxidase and placed in the oven at 55°C for 2 hours (using method adapted from Vasylieva *et al.*[6,7]). Finally the biosensors were coated with a polyurethane diffusion-limiting film to extend their dynamic range. The biosensors were positioned in a poly(dimethylsiloxane) (PDMS) microfluidic chip[8] as described elsewhere.[3] Biosensors were controlled using in-house potentiostats and a PowerLab 8/35, controlled by LabChart Pro (ADInstruments).

High temporal resolution analysis

Figure S1 illustrates the concept that collection and analysis flow rates can be optimised independently using this methodology. In the example described, microdialysis is carried out at 5 μl/min and dialysate is collected in storage tubing. Subsequent analysis takes place at 1 μl/min, effectively giving a reading every 12 s in real time at the expense of the analysis now taking 5 hours.

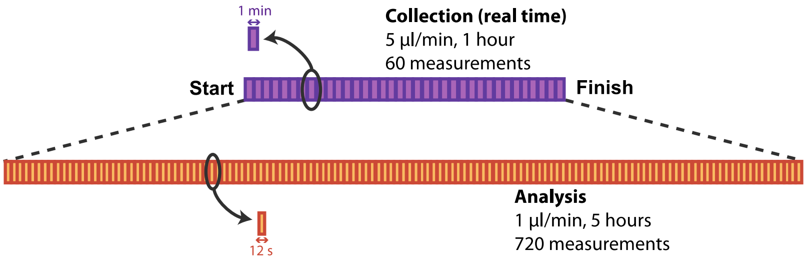

Figure SI. Schematic representation of temporal resolution at different flow rates. In this example we imagine an analysis time of 1 min and a sample loop of 0.5 µl. If samples are collected and analysed at 5 µl/min this would give 60 measurements in 1 hour of dialysate (assuming complete loop filling with 2x loop volume). If instead the analysis flow rate is slowed down to 1 µl/min this would give 720 measurements per hour of collected dialysate.

Axial molecular diffusion

Taylor dispersion due to passive diffusion during static storage is negligible; a molecule in solution diffuses a distance of appoximately $\sqrt{2Dt}$ (where t is time in seconds and D is the diffusion coefficient. For example, it would take a particle 5×10^{6} s (equivalent to nearly 2 months) to diffuse 10 cm (assuming D is 1×10^{-5} cm^{2}s^{-1} at room temperature), equivalent to 1.1 µl volume in FEP tubing (0.12 mm ID).

Microdialysis probe pressure limits

Kiritsis measured the ratio of fluid velocity through the membrane as a function of the pressure gradient across the membrane. This quantity is called L_p. It is related to components of Darcy's Law as shown in Eq. 1.

$$L_p = \frac{v}{\Delta P} = \frac{\kappa}{L\eta} \quad (1)$$

Because the membrane expands slightly under pressure, L_p increases as pressure increases. The following calculations are for $L_p = 0.9 \times 10^{-12}$ m/(Pa.s), which is the value at $\Delta P = 50$ kPa (~0.5 atm). The probe used was typical, namely with an inside diameter of 200 µm and a length of 2 mm. As flow rate is just the surface area, A, through which fluid flows multiplied by the velocity,

$$Q = vA = A\Delta P L_p \quad (2)$$

For $L_p = 0.9 \times 10^{-12}$ m/(Pa.s), $\Delta P = 50$ kPa and the area of the probe's membrane (1.3×10^{-6} mm^2), we find that the flow rate is 3 nl/min. Under many circumstances this is negligible.

References

1 M. L. Rogers, P. A. Brennan, C. L. Leong, S. A. N. Gowers, T. Aldridge, T. K. Mellor and M. G. Boutelle, *Anal. Bioanal. Chem.*, 2013, 405, 3881–3888.
2 K. Hamaoui, S. Gowers, S. Damji, M. Rogers, C. L. Leong, G. Hanna, A. Darzi, M. Boutelle and V. Papalois, *J. Surg. Res.*, 2015, 200, 332–345.
3 M. L. Rogers, C. L. Leong, S. A. Gowers, I. C. Samper, S. L. Jewell, A. Khan, L. McCarthy, C. Pahl, C. M. Tolias, D. C. Walsh, A. J. Strong and M. G. Boutelle, *J. Cereb. Blood Flow Metab.*, 2016, 0271678X16674486.
4 S. A. N. Gowers, V. F. Curto, C. A. Seneci, C. Wang, S. Anastasova, P. Vadgama, G. Z. Yang and M. G. Boutelle, *Anal. Chem.*, 2015, 87, 7763–7770.
5 B. A. Patel, M. Rogers, T. Wieder, D. O'Hare and M. G. Boutelle, *Biosens. Bioelectron.*, 2011, 26, 2890–6.
6 A. Viggiano, S. Marinesco, F. Pain, A. Meiller and H. Gurden, *J. Neurosci. Methods*, 2012, 206, 1–6.
7 N. Vasylieva, B. Barnych, A. Meiller, C. Maucler, L. Pollegioni, J.-S. Lin, D. Barbier and S. Marinesco, *Biosens. Bioelectron.*, 2011, 26, 3993–4000.
8 M. Rogers, C. Leong, X. Niu, A. de Mello, K. H. Parker and M. G. Boutelle, *Phys. Chem. Chem. Phys.*, 2011, 13, 5298–5303.
